# Outcome of Laparoscopic Appendectomy: A Retrospective Study From a Local Secondary Referral Hospital

**DOI:** 10.7759/cureus.73791

**Published:** 2024-11-16

**Authors:** Haytham Alarfaj, Mohammed S Bu Bshait

**Affiliations:** 1 Department of Surgery, College of Medicine, King Faisal University, Al Ahsa, SAU

**Keywords:** appendectomy, appendicitis, laparoscopy, outcome, secondary referral settings

## Abstract

Introduction

Laparoscopic appendectomy is currently considered the operation of choice for patients with suspected acute appendicitis. This study aimed to assess the safety and feasibility of laparoscopic appendectomy in the setting of a secondary referral hospital.

Methods

A retrospective cohort study was conducted from January 2021 to December 2023. Records of patients older than 14 years of age who underwent appendectomy were retrieved. Patients were divided according to the type of technique used into two groups: group I (G1) consisted of patients who underwent laparoscopic appendectomy and group II (G2) included those with an open appendectomy. Patients who underwent interval appendectomy or incidental to other procedures were excluded. The two groups were compared in terms of patient demographics, pathological findings, operative time, postoperative course, and outcome.

Results

Laparoscopic appendectomy was performed in 101 patients while open appendectomy was done in 121 patients. There were no statistical differences between both groups regarding operative time, blood loss, time for oral intake resumption, hospital stay, or postoperative complications. Despite being not statistically significant, surgical site infection was lower in G1 as compared to G2 (3% versus 8.3%, p=0.09). Narcotic use was significantly less following laparoscopic appendectomy. In the late settings cases of G1, there was a relatively improved mean operative time (32.7 ±18.3) when compared to early settings appendectomy (62.4±26.3), which was statistically significant (*P*=0.001).

Conclusion

Laparoscopic appendectomy is safe, feasible, and adoptable even by junior staff. Therefore, it could be applicable in settings of secondary referral hospitals as an initial line of management when performed by an expert surgeon or trainee under the supervision of seniors.

## Introduction

Appendicitis is the most common intraabdominal condition requiring emergency surgery, with about a 6-8% lifetime risk [1]. Appendectomy, since its introduction by McBurney in 1894 [2], has been the treatment of choice for acute appendicitis. McBurney’s incision represented the gold standard for acute appendicitis until 1983 when Semm introduced laparoscopic appendectomy [3]. As the operative techniques were refined, the indications have extended to patients with suspected appendicitis [1,4,5]. Unlike laparoscopic cholecystectomy, laparoscopic appendectomy has struggled to prove its superiority over the open technique [6]. However, nowadays the number of laparoscopic appendectomies has progressively increased since it has been demonstrated to be a safe procedure, with excellent cosmetic results and it allows shorter hospitalization and quicker and less painful postoperative recovery. In contrast to the trend of laparoscopic appendectomy being the surgery of choice for appendicitis [7,8], there was reluctance to establish it in our institute. That was probably due to the trepidation to overcome a simple, quick, and safe procedure with an expective mortality. Furthermore, as a secondary referral setting, hospital policies were initially restricted toward such intervention to ensure the patient's safety. These included a prohibition of performing laparoscopic appendectomy at night and the necessity of a consultant to perform it. However, the inclusion of laparoscopic appendectomy in the surgical training curriculum along with the evolution of trained staff has recently expanded the application of laparoscopic appendectomy in our hospital.

The current study aimed to assess the safety and feasibility of laparoscopic appendectomy in our institute of secondary referral settings.

## Materials and methods

**Figure 1:**
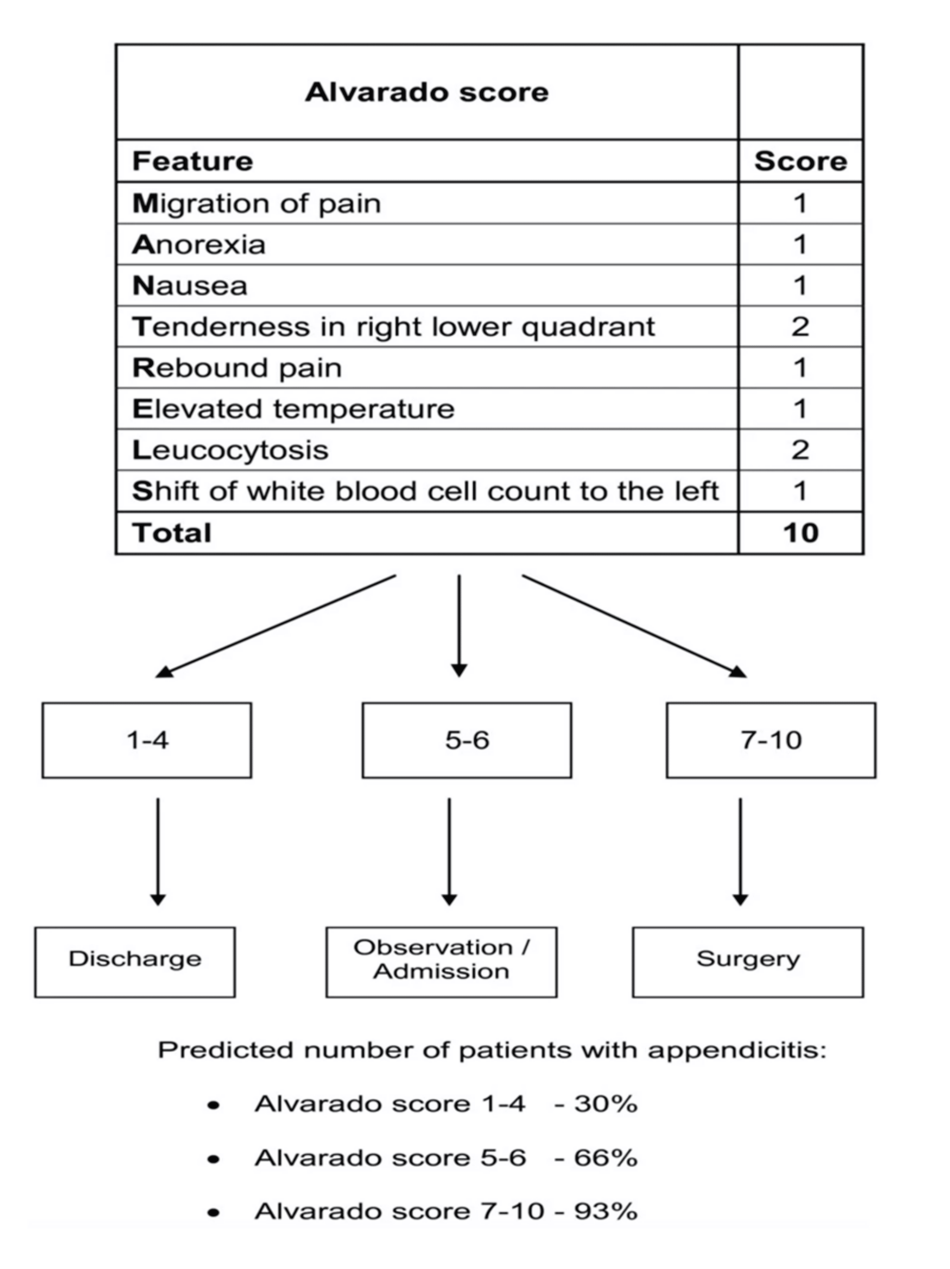
Scoring parameters and interpretation of the Alvarado score used for the diagnosis of acute appendicitis Source: [11]

This was a retrospective cohort study conducted over three years from January 2021 to December 2023 to navigate the outcome of the emerging laparoscopic appendectomy in comparison to open conventional appendectomy at a local secondary referral hospital in Saudi Arabia. Medical records for patients who underwent appendectomy for acute appendicitis at Prince Saud bin Jalawi Hospital, Al-Ahsa, Saudi Arabia, during the study period were retrieved. All appendectomy patients aged 14 years or above were included in the study. Exclusion criteria comprised those who were below the age of 14, who underwent interval appendectomy, or in cases where appendectomy was performed incidentally to other procedures. The preoperative indications of appendectomy included patients with clinically suspected acute appendicitis and/or confirmed by imaging studies. In addition, laparoscopic appendectomy was particularly selected for those patients with an equivocal clinical presentation concerning for acute appendicitis or inconclusive investigations, aiming for diagnostic exploration and management accordingly. All patients were clinically diagnosed with acute appendicitis by applying the Alvarado scoring system [9]. However, equivocal findings when applying the Alvarado score were reported in 12 (5.4%) patients. In all these patients, harmonic ultrasound was performed as previously described [10]. Yet, in 8 out of them (66.7%), it was conclusive. However, the results were not conclusive in the remaining 4 (33.3%) who underwent multidetector computed tomography scanning. Figure 1 shows the scoring parameters and interpretation of the Alvarado score used for the diagnosis of acute appendicitis [11].

A total of 246 patients' records were initially screened. Based on the exclusion criteria, 24 patients were excluded. The total number of the included patients was 222. Patients were divided according to the type of the applied surgical technique into two groups: Group I (G1) consisted of patients who underwent laparoscopic appendectomy (101 patients) and Group II (G2) consisted of those with an open appendectomy (121). The two groups were compared with regard to patients' demographics, antibiotic and analgesia utilization, operative findings, duration of surgery, hospital stay, postoperative course, and outcome.

To assess the evolution of our experience with laparoscopic appendectomy, G1 was subdivided into early and late settings. Early settings represent the cases performed within the first six months of the study period (32 patients) while the subsequent cases were determined as late settings (96 patients). The subdivided groups were compared with regard to operative and postoperative courses and outcomes.

The primary outcome of the current study was to determine the safety of laparoscopic appendectomy. Secondary outcomes were the duration of surgery, time for oral intake resumption, hospital stay, and the differences in laparoscopic appendectomy outcomes as our experience progressed.

The ethical issues were designed in accordance with our institution's guidelines. The study protocol was reviewed and approved by the research ethics committee of the College of Medicine, King Faisal University (Approval No. REC2017-3-14, on 11-02-2024).

Statistical analysis

Upon data completion and validation, data were transported from Microsoft Excel (Microsoft Corporation, Redmond, WA, US) to the statistical software package SPSS Ver. 25 (IBM Corp., Armonk, NY, US) for statistical analysis. Both descriptive and analytic inferential statistics were conducted. A P-value of ≤ 0.05 was accepted as a significance level for all statistical tests. Categorical variables were presented as counts and proportions (%) and continuous variables were presented as mean ± standard deviation. In a univariate analysis for comparison and correlation between variables of interest versus different categorical variables, a chi-square as well as an independent t-test were applied. Multivariate analyses were conducted as well, where the odds ratio with a significance level and a 95% confidence interval (CI) were reported.

## Results

Total number of patients who underwent appendectomy during the period from January 2021 to December 2023 was 246. After applying the exclusion criteria, the total number of patients included in the study was 222. Males were 119 and females were 103 with a male-to-female ratio of 1.2:1. Their mean age was 25.5±10.3. About 37 patients (17%) had associated co-morbidities. Twenty (54%) had gynecological and sickle cell diseases. The gynecological disorders were polycystic ovaries in 10 cases while 4 patients had ovarian cysts that were not complicated. On the other hand, six patients were suffering from sickle cell disease, which is a type of hemolytic anemia commonly seen in the local community; yet, they were controlled with no history of recurrent vaso-occlusive crises. Other reported co-morbidities were diabetes and hypertension, which were found in 10 patients. According to the method of operation, 101 underwent laparoscopic appendectomy (G1) while the remaining 121 patients underwent open appendectomy (G2) (Table 1).

**Table 1:** Descriptive analysis for patients’ socio-demographic characteristics (n=222) * Results are expressed as mean ± standard deviation, number, and percentage (%). n: Number

Factor	Results*
Age in years	25.5 ± 10.3
Gender	
Male	119 (53.6%)
Female	103 (46.4%)
Method of operation	
Laparoscopic	101 (45.5%)
Open	121 (54.5%)
Associated disease	
None	185 (83.3%)
Sickle cell disease	06 (02.7%)
Ovarian cyst	14 (06.3%)
Diabetes and/or hypertension	10 (04.5%)
Others	07 (03.2%)
Length of stay (days)	03.7±1.9

Comparing the patients after dividing them into the corresponding groups revealed interesting findings regarding different variables. There was no notable difference in regard to patients' age and body mass index among both groups. Also, there was no significant difference between G1 and G2 related to using antibiotics, which revealed that almost all patients in both groups had preoperative and postoperative antibiotics. Those who showed no or mild inflammation in the appendix intra-operatively did not receive postoperative antibiotics. Intra-operative reported findings in regards to complicated (gangrenous or perforated) appendicitis or uncomplicated appendicitis didn't differ statistically between both groups. Complicated appendicitis was found in eight patients within G1 compared to seven patients in G2. The mean length of hospital stay in G1 was 3.6±1.7 while it was 3.8±2.1 in G2 with no statistical significance (p=0.455). Despite the notable increase in operative time in G1, there were no significant differences regarding the operative time between the two groups (36.6±17.1 vs 28.9±15.8, p=183). However, during the initial implementation of the laparoscopic appendectomy procedure, four patients underwent conversion from laparoscopy to open appendectomy as injuries to internal organs were anticipated in one patient plus difficult procedures in the remaining cases. This has led to an increased operative time of up to 75 minutes. Nevertheless, with the improvement of the learning curve, the shortest operative time was recorded to be 15 minutes. The mean time of food resumption was almost similar: 19.8±9.3 and 19.4±9.7 hours for G1 and G2, respectively. Only 8% of patients in G1 required narcotic analgesia compared to 18.2% in G2 (p=0.026). Postoperative complications were reported in 7 (6.9%), and 14 patients (11.6%) among G1 and G2, respectively, with no statistical significance (p=0.239). The postoperative complications among G1 were as follows: surgical site infection was found in three patients, paralytic ileus in three patients, and intra-abdominal abscess in one patient. Among G2, a surgical site infection was reported in 10 patients, with intra-abdominal abscess and 1 patient with enterocutaneous fistula. Despite the lower surgical site infection rate in G1 compared to G2 (3% Vs 8.3%, p=0.094), this observation was not statistically significant (Table 2). Among those patients who suffered from postoperative complications, the histopathology of the removed appendix showed pyogenic inflammation. Moreover, it was noted that 9 out of the total 13 patients (69%) had a BMI that ranged between 29 to 35 with a mean of 31±4. This may indicate that being overweight was a significant factor for wound-related complications despite the used technique.

**Table 2:** Association between method of operation and patients' characteristics (n=222) Results are expressed as mean ± standard deviation, number, and percentage (%). n: Number. ∞ The P-value has been calculated using the chi-square test and independent t-test. ** Significant value p <0.05.

Factor	Method of operation		P-value ^∞^
	Laparoscopic (n=101)	Open (n=121)	
Age	25.4±6.2	23.9±9.6	0.133
Body mass index	23.2±7.7	22.7±8.3	0.205
Length of stay (days)	03.6 ± 01.7	03.8 ± 02.1	0.455
Preoperative antibiotic:			
No	03 (03.0%)	03 (02.5%)	0.822
Yes	98 (97.0%)	118 (97.5%)	
Postoperative antibiotic:			
No	01 (01.0%)	05 (04.1%)	0.151
Yes	100 (99.0%)	116 (95.9%)	
Postoperative analgesia:			
No	02 (02.0%)	04 (03.0%)	0.544
Yes	99 (98.0%)	117 (96.7%)	
Type of analgesia:			
Non-narcotic	93 (92.1%)	99 (81.8%)	0.026 *
Narcotic	08 (07.9%)	22 (18.2%)	
Duration of surgery (minutes)	36.6±17.1	28.9±15.8	0.183
Blood loss (millimeter)	09.6 ± 10.2	10.7 ± 10.8	0.441
Time for oral intake resumption (hour)	19.8 ± 09.3	19.4 ± 09.7	0.758
Complications:			
No	94 (93.1%)	107 (88.4%)	0.239
Yes	07 (06.9%)	14 (11.6%)	
Surgical site infection:			
No	98 (97.0%)	111 (91.7%)	0.094
Yes	03 (03.0%)	10 (08.3%)	
Complicated appendicitis:			
No	93 (92.1%)	114 (94.2%)	0.528
Yes	08 (07.9%)	07 (05.8%)	

After subdividing G1 into early and late settings, the operative and postoperative courses and outcomes were compared. Late-setting cases showed a significant decline in operative time compared to early settings (62.4±26.3 vs 32.7±18.3, p=0.001). All laparoscopic appendectomy were performed using the endoloop technique for the sake of cost-effectiveness and safety. The severed appendix was put in a bag to be extracted from inside the abdominal cavity. The incidence of postoperative complications among early settings cases was found to be relatively higher (12.5%) compared to 4.3% in the late settings’ laparoscopic appendectomies. However, this finding was not statistically significant. Furthermore, early and late settings didn’t show differences in regard to intra-operative blood loss, time to oral intake resumption, and hospital stay (Table 3).

**Table 3:** Comparison between the outcomes of laparoscopic appendectomy in early and late settings (n=101) Results are expressed as mean ± standard deviation, number, and percentage (%). n: Number. ∞ The P-value has been calculated using the chi-square test and independent t-test. ** Significant value p <0.05.

Factor	Laparoscopic appendectomy		P-value ^∞^
	Early settings (n=32)	Late settings (n=69)	
Length of stay (days)	03.9 ± 01.9	03.4 ± 01.3	0.253
Duration of surgery (minutes)	62.4 ± 26.3	32.7 ± 18.3	<0.001**
Blood loss (millimeter)	09.8± 10.2	8.9 ± 10.8	0.411
Time for oral intake resumption (hour)	20.1 ± 08.6	19.2 ± 09.7	0.659
Complications:			
No	28 (87.5%)	66 (95.7%)	0.081
Yes	04 (12.5%)	3 (4.3%)	

## Discussion

The current study explored the safety and feasibility of laparoscopic appendectomy in the settings of the secondary referral hospital where this intervention has emerged. We found that laparoscopic appendectomy is safe and readily adoptable if it has been implemented through the existence of both equipment and expert personnel. Despite being relatively new to our settings, it showed comparable results to the existing literature in terms of intervention outcomes and the incidence of complications.

Management of acute appendicitis still poses a challenge for surgeons despite being an old health problem [12]. Many authors have advocated surgery as well as conservative management for this pathology [13-15]. Conventional appendectomy has been practiced for many years. Nevertheless, this technique usually may not entail an accurate clinicopathological diagnosis. In the past three decades, laparoscopy has been advocated as a feasible standard modality in diagnosis and surgical removal of acutely inflamed appendix [1]. Recently, about 80% of appendectomies in the USA have been performed laparoscopically [7].

We currently have studied and compared open appendectomy versus laparoscopic appendectomy in 222 patients. Their mean age was 25.5±10.3; this is relatively comparable to previously published data of the patients' mean ages of 23 and 25 for open and laparoscopic appendectomy, respectively [16,17]. In the current study, the male-to-female ratio was 1.2:1, simulating what was previously published [16-21]. However, it was contradictory to other data that showed a higher incidence of females than males as regards acute appendicitis [8,22,23]. The reason for this discrepancy, especially among the laparoscopically managed group may be attributed to the fact that laparoscopy may add to the diagnosis of more cases of females' acute appendicitis that might have been previously neglected and misdiagnosed. Open appendectomy accounted for 54% of our study cases while laparoscopy was done in 46%. These data simulate few published literature reports [6,17,22], although many have demonstrated a higher incidence of laparoscopic rather than open appendectomy [8,16-21]. This may be explained by the fact that laparoscopic appendectomy was relatively new in our session and most of the surgeons in our secondary referral hospital were not familiar with the technique, especially within the initial time of the study. This may be justified by the longer mean operative time in our early settings. Most of our cases had no associated co-morbidities, yet, some did show sickle cell anemia. The prevalence of this disease in our area is about 145 cases per 10.000 population and is considered an endemic trait [24]. Therefore, they might present with symptoms mimicking those of acute appendicitis in certain conditions. All these cases underwent laparoscopic procedures aiming at both diagnosis and treatment. This highlighted the role of laparoscopy as an initial tool in dealing with those cases. However, the number of sickle cell disease patients included in this cohort was limited. Overall, laparoscopic appendectomy was done in these patients, even those who did not show inflammation. Among these cases, a thorough laparoscopic examination of the abdomen was performed to detect any other causes of the clinical presentation. In two cases, colonic edema and inflammation of the wall were detected raising the diagnosis of colitis. There were no cases of Meckle's diverticulitis detected laparoscopically. Other co-morbidities were also reported in the series; including diabetes mellitus and hypertension, as well as other co-morbidities as previously published in the literature [23]. Most patients had postoperative antibiotics contradicting previously published reports [18,23,25,26]. The reason for this may be attributed to our low experience curve in laparoscopic procedures with excessive tissue manipulation.

The total length of hospital stay in our series was collectively 3.7±1.8, which is relatively higher compared to others, who even performed a laparoscopic appendectomy on an outpatient basis [27,28]. Two reasons may be explaining this condition; the first is the higher number of open appendectomy compared to laparoscopy. The second reason is due to the lack of experience in laparoscopic appendectomy especially the early cases. The same concept can be applied to the excessive use of postoperative analgesia. However, most laparoscopic appendectomy patients did not need any narcotic analgesia. This observation was significant when compared with the open appendectomy group (P=0.026). This coincides with other previously published data and supports the fact that laparoscopic appendectomy minimizes postoperative pain [21,29,30]. Despite the relatively longer operative time which was essentially in the earlier cases, the mean operative time became comparable to what has been published [16,31,32]. This notion also affected the resumption of oral feeding among our patients, which was relatively high compared to other reports [6,21]. We, however, reported very limited cases of complications as others [27,33-36]. Furthermore, there was an observed improvement in operative time and incidence of postoperative complications as our experience in laparoscopic appendectomy evolved. This may be related to the meticulous training of our staff and their experience as competent surgeons.

This study has potential limitations. These include the relatively small sample size and the retrospective nature of the study, which may have weakened the methodology and contained a lot of bias. The study lacked histological data and correlations. Moreover, It was performed in a single secondary referral hospital. Hence, the results might not reflect a realistic image of the notion of feasibility and safety of laparoscopic appendectomy in such settings. Further well-designed, prospective, multicentric, and adequately powered studies need to be done to explore the outcomes of laparoscopic appendectomy more explicitly. 

## Conclusions

It may be concluded that laparoscopic appendectomy is feasible, safe, and adoptable in a secondary referral hospital by junior staff. It has the merit of being diagnostic as well as therapeutic with a limited number of complications. As laparoscopic appendectomy would be available as regards the instruments and personnel in secondary referral settings, it would be recommended to be more widely adopted for cases of acute appendicitis.
